# Resveratrol ameliorates osteogenic differentiation, calcification, and apoptosis of VSMCs through regulating JNK/Bax signaling

**DOI:** 10.3389/fphar.2025.1631039

**Published:** 2025-09-01

**Authors:** Menglin Hou, Hui Wang, Junmei Cheng, Xuyan Duan, Min Ren, Yong Lu

**Affiliations:** ^1^ Department of Basic Medical, Heze Medical College, Heze, Shandong, China; ^2^ Department of Central Laboratory, Heze Medical College, Heze, Shandong, China

**Keywords:** resveratrol, calcification, JNK/Bax signaling, vascular smooth muscle cells, apoptosis

## Abstract

**Introduction:**

Vascular calcification involves pathological mineralization in the vascular wall, which is characterized by the transformation of vascular smooth muscle cells (VSMCs) from a contractile phenotype to a synthetic phenotype. VSMCs undergoing apoptosis were found in vascular calcified plaques. However, the regulatory role of resveratrol in vascular calcification via VSMC apoptosis modulation remains unclear.

**Methods:**

Rat VSMCs were cultured in calcifying medium (CM) to induce calcification, and treated with resveratrol, the JNK inhibitor SP600125, or the JNK activator anisomycin. Calcium deposition was assessed via alizarin red staining and quantitative calcium content assays. Alkaline phosphatase (ALP) activity, mRNA and protein levels of osteogenic markers, and apoptosis were evaluated. Molecular docking was performed to predict resveratrol-JNK binding. In vivo, vitamin D_3_-induced vascular calcification in mice was treated with resveratrol, and aortic calcification was analyzed via von Kossa and alizarin red staining.

**Results:**

In CM-induced rat VSMC calcification, resveratrol treatment effectively attenuated the calcification of VSMCs, as evidenced by reduced calcium content, ALP activity, and osteogenic markers including Runx2, BMP2, and Osterix levels. Furthermore, resveratrol treatment significantly suppressed TUNEL-positive cell proportions and caspase-3 activity in CM-treated VSMCs. Mechanistically, resveratrol treatment blocked JNK/Bax activation by reducing the p-JNK and Bax levels in CM-treated VSMCs. The JNK inhibitor SP600125 markedly reduced calcification, downregulated osteogenic markers, and inhibited apoptosis in CM-treated VSMCs. JNK activation reversed resveratrol’s anti-calcification and anti-apoptotic effects. In vitamin D_3_-induced calcification models, resveratrol significantly reduced vascular calcification and osteogenic differentiation.

**Discussion:**

Resveratrol exerted an inhibitory effect on VSMC calcification, osteogenic differentiation, and apoptosis through the inhibition of the JNK/Bax signaling pathway.

## 1 Introduction

Vascular calcification is defined as the pathological phenomenon of abnormal deposition of calcium and phosphorus crystals in the vascular wall. It is defined as a complication of chronic kidney disease, diabetes, aging, atherosclerosis, and other diseases (Shanahan et al., 2011; Wilson et al., 2001). Additionally, vascular calcification primarily affects arterial vessels, contributing to increased arterial stiffness and diastolic and systolic dysfunction, and it further causes a variety of cardiovascular complications (Lacolley et al., 2017; Lanzer et al., 2021). Historically, vascular calcification is a passive process of calcium phosphate deposition in vascular cells and the extracellular matrix due to the imbalance of calcium metabolism in the body. Currently, vascular calcification is increasingly recognized as an active process regulated by multiple pathways analogous to bone formation (Chen et al., 2020; Hodroge et al., 2017; Villa-Bellosta and Egido, 2017). Calcification is characterized by the transformation of vascular cells to an osteoblast-like phenotype, upregulation of various osteogenic marker proteins, and increased intracellular alkaline phosphatase activity. However, the molecular regulation of vascular calcification remains to be elucidated.

Resveratrol (Res) is a non-flavonoid polyphenolic compound found in plants belonging to the grapevine, lily, and legume families (Shukla and Singh, 2011). As a natural and non-toxic compound, Res exhibits diverse biological activities, including cardiovascular protection, anti-tumor, anti-inflammatory, anti-oxidation, and estrogen mimicking (Sadruddin and Arora, 2009). Furthermore, Res acts as a natural activator of SIRT1 gene expression, which promotes cell survival and protects against apoptosis (Poussier et al., 2005; Yeung et al., 2004). Res can be rapidly absorbed, distributed, and metabolized by the human body after oral administration (Bishayee, 2009). Studies have demonstrated that Res significantly improved endothelial cell damage, atherosclerotic plaque formation, cardiovascular remodeling, and other cardiovascular diseases (Bonnefont-Rousselot, 2016; Guo et al., 2022; Li et al., 2019). As an important element in cardiovascular diseases, vascular smooth muscle cells (VSMCs) are key targets for Res, which inhibits VSMC proliferation, migration, and senescence (Lin et al., 2014; Uhrin et al., 2018). However, whether Res has a direct intervention effect on the phenotypic changes of VSMCs and vascular calcification is still not fully known.

In this study, we employed the calcification medium (CM) and vitamin D_3_ to induce VSMC calcification and mouse vascular calcification, respectively, and used Res to observe its effect on whole vascular calcification, thereby elucidating its molecular mechanism in inhibiting vascular calcification. Notably, these findings offer novel insights into the prevention and treatment of vascular calcification.

## 2 Materials and methods

### 2.1 Cell culture

VSMCs were isolated from aortic arteries of male Sprague–Dawley (SD) rats (150 g–180 g) using the explant method. VSMCs were cultured in DMEM [10% FBS, penicillin (100 units/mL), and streptomycin (100 mg/mL)] at 37 °C in an incubator. VSMCs between passages 5 and 8 were used for the experiments. VSMC calcification was induced by the CM [DMEM, β-glycerophosphate (β-GP, 10 mM), and CaCl_2_ (3 mM)]. After the cells were adhered overnight, VSMCs were treated with Res (10 μM; Sigma, St. Louis, MO) in the CM for 10 days (Li et al., 2024; Takemura et al., 2011). The C-jun N terminal kinase (JNK) inhibitor (SP600125, 10 μM; Sigma) or activator (anisomycin, 100 ng/mL; Sigma) was added to the medium 1 h before Res treatment for use in the experiments (Liu et al., 2021; Zhang et al., 2015).

### 2.2 Cell viability assays

VSMCs in the logarithmic growth stage were inoculated into a 96-well plates (1,000 cells per/well), with five wells in each group. After 24 h of cell inoculation and adherent growth, Res was administered at the final concentrations of 0, 10, 20, 25, 50, 75, and 100 µM (Li et al., 2024). After 24 h, 10 µL of CCK-8 solution was added to each well and incubated in the dark for 30 min. The absorbance (A) value was measured at 450 nm.

### 2.3 Determination of cell calcification

After fixed in 4% formaldehyde for 10 min in 6-well plates, VSMCs were treated with 2% alizarin red (pH 4.2) for 5 min. Then, excess dye was removed by deionized water, and an inverted phase contrast microscope was used to visualize staining. Alizarin red dye was eluted with 10% formic acid and quantified by spectrophotometry. As previously described, the calcium content and alkaline phosphatase (ALP) activity was measured (Hou et al., 2016; Liao et al., 2013; Yan et al., 2011). VSMCs were washed with phosphate-buffered saline (PBS) and extracted with 0.6 N HCl for 24 h. The protein concentration was quantified using BCA protein assay (Pierce, United States). The calcium content was normalized to protein concentration and expressed as µg/mg protein. For ALP activity analysis, VSMCs were harvested with 0.1% Triton X-100 in PBS. p-NPP (180 µL) substrate was added to the protein samples, and the reaction was incubated for 15 min at 37 °C. To stop the reaction, NaOH (3 M) was added to the mixture. The absorbance was then measured at 405 nm, and the ALP activity was presented as nmol/mL p-nitrophenol converted per microgram of protein per minute.

### 2.4 Western blot analysis

Total protein was extracted from VSMCs, and the protein concentration was measured using a BCA protein assay kit (Pierce, United States). Equal amounts of protein samples were loaded and separated by 5% SDS-PAGE and then transferred to nitrocellulose membranes (Bio-Rad, United States). The membranes were then blocked with 5% non-fat dried milk for 1 h, followed by incubation with primary antibodies including Bax (1:1,000, 2772), JNK (1:1,000, 9252), p-JNK (1:1,000, 9255), and β-actin (1:3,000, ab32572) from Cell Signaling Technology (Beverly, MA, United States), along with Runx2 (1:500, SAB1403638, Millipore, United States) and Osterix2 (1:1,000, ab209484, Abcam, United Kingdom). Then, membranes were incubated with secondary antibodies (1:1,000, 5125, Cell Signaling, United States). SuperSignal West Pico Chemiluminescent Substrate (Pierce, United States) was used to detect the protein signals.

### 2.5 Quantitative real-time PCR

TRIzol reagent (Invitrogen, United States), a reverse transcription kit (Takara Company, China), a StepOne Real-Time PCR system (Applied Biosystems, United States), and SYBR Green mixture were utilized for PCR. PCR primers were as follows: Runx2 (forward): GCC GGG AAT GAT GAG AAC TA, Runx2 (reverse): GGA CCG TCC ACT GTC ACT TT; BMP2 (forward): GTT TGG CCT GAA GCA GAG AC, BMP2 (reverse): CTC GAT GGC TTC TTC GTG AT; β-actin (forward): TGT​CAC​CAA​CTG​GGA​CGA​TA, β-actin (reverse): GGG GTG TTG AAG GTC TCA AA; and Osterix (forward): TCT CCA TCT GCC TGA CTC CT, Osterix (reverse): GGG GCT GAA AGG TCA GTG TA. Housekeeping gene β-actin and the comparative Ct method were used to determine the target gene expression in the experimental group.

### 2.6 Cell apoptosis assay

The One-Step TUNEL Apoptosis Assay Kit (Beyotime, Shanghai, China) and Caspase-3/CPP32 Colorimetric Assay Kit (BioVision) were used to detect cell apoptosis according to the manufacturers’ instructions. After treatment, VSMCs were fixed in 4% (w/v) paraformaldehyde at 4 °C, followed by incubation with terminal deoxynucleotidyl transferase (TdT) for 1 h. The cell nuclei were stained with DAPI. The TUNEL-positive apoptotic VSMCs were detected using a fluorescence microscope (Olympus). In addition, VSMCs were lysed, and the protein concentration was measured using a BCA protein assay kit (Pierce, United States). Protein (100 µg) was mixed with cell lysis buffer (50 µL) and reaction buffer (50 µL). Then, 5 µL of 4 mM DEVD-qNA substrate was added and incubated at 37 °C for 2 h. The activity of caspase 3 was detected by a microplate reader at 405 nm.

### 2.7 Immunofluorescence staining and immunohistochemistry staining

VSMCs were incubated with the antibodies Runx2 (1:100, SAB1403638, Millipore), p-JNK (1:200, 9255, Cell Signaling, United States), and Bax (1:200, 2772, Cell Signaling, United States), followed by staining with FITC-conjugated secondary antibody. VSMCs were then double-stained with DAPI for visualizing the nuclei and viewed with a Nikon Eclipse 80iEpi-fluorescence microscope equipped with a digital camera (DS-Ri1, Nikon).

### 2.8 Molecular docking

Docking compound Res (compound CID: 445154) was obtained from the PubChem database (https://pubchem.ncbi.nlm.nih.gov/), and JNK (ID: P49185) was obtained from the UniProt database (https://www.uniprot.org/). The processing and optimization of the molecular docking process were performed by the Glide module in the Schrodinger Maestro software. For screening in the Glide module, the prepared receptors were imported, the protein protoligand was selected as the binding site of the protein, and the box size was set to 10Å × 10Å × 10Å. The complexes of the JNK proteins and Res were visualized by PyMOL 2.1.

### 2.9 Animals and treatment

All animal experimental procedures in this study were performed in accordance with the requirements of the Ethics Committee for Heze Medical College. The vascular calcification model was established in 8–10-week-old male C57BL/6 mice (weighing 20 g–25 g). The mice were randomly divided into a vehicle group (n = 6), a vitamin D_3_ group (Vit D group, n = 6), and a vitamin D_3_ + resveratrol group (Vit D + Res group, n = 6). Briefly, the mice received subcutaneous injection of vitamin D_3_ (cholecalciferol, 5 × 10^5^ IU/kg/day) once a day for 3 consecutive days, as previously described (Bhat et al., 2020; Zeng et al., 2021; Zhang et al., 2021). In the vehicle control group, the mice were treated with an injection of matched vehicle (5% v/v ethanol). In the vitamin D_3_ + resveratrol group (Vit D+ Res group), the mice received the same vitamin D_3_ regimen. Starting 1 day after vitamin D_3_ injection, these mice were co-treated with Res (50 mg/kg/day) by intraperitoneal injection for 7 consecutive days. All mice were sacrificed at the end of the experimental period, and the aortic vessels were harvested for subsequent experiments.

### 2.10 Vascular von Kossa staining

Frozen sections of the aorta were allowed to rewarm at room temperature for 2 h and then were hydrated in 1× histochemical PBS. A sufficient amount of 5% AgNO_3_ solution was applied onto the vascular tissue to completely cover the tissue and then irradiated under UV light for 30 min–60 min. After discharging the AgNO_3_ solution, the sections were washed with distilled water and 5% Na_2_S_2_O_3_ solution for 2 min, respectively. After staining with the hematoxylin solution, the sections were successively dehydrated with 70%, 80%, 90%, 95%, 100%, and 100% ethanol and then made transparent with xylene. The sections were sealed with neutral gum and baked to dryness in an incubator at 37 °C.

### 2.11 Statistical analysis

All data were expressed as the mean ± SD and analyzed using the software package SPSS 17.0. Statistical differences between the two groups were analyzed by Student’s t-test, and the differences between more than two groups were compared by one-way ANOVA. A value of p < 0.05 was considered statistically significant.

## 3 Results

### 3.1 Elevated calcium and phosphate levels promote osteogenic differentiation and calcification of rat VSMCs

**FIGURE 1:**
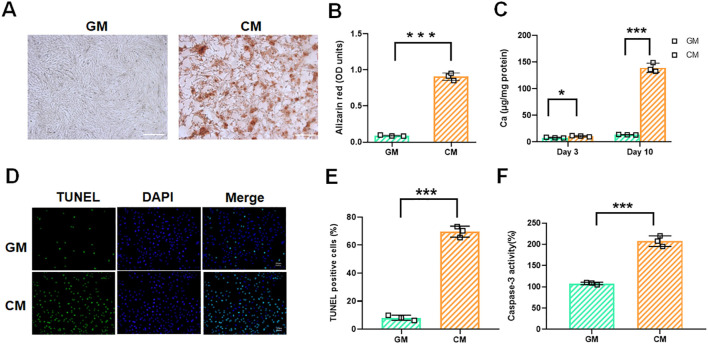
Calcification and apoptosis in VSMCs after exposure to CM. VSMCs were treated with GM or CM, respectively. **(A,B)** Alizarin red staining and quantification of alizarin red analysis of calcium deposition in VSMCs. Scale bar = 200 μm. **(C)** Calcium content analysis of the results of calcium deposition in VSMCs. **(D–F)** TUNEL staining and caspase-3 activity assay analysis of cell apoptosis in VSMCs. Scale bar = 20 μm *P < 0.05 and ***p < 0.001.

To investigate vascular calcification, we established a cell calcification model using CM to treat VSMCs. Alizarin red staining confirmed the successful induction of the cell calcification model, with visible calcium deposition (Figures 1A, B). Calcium concentration in CM-treated VSMCs significantly increased over time (Figure 1C). VSMCs exhibited increased ALP activity after CM treatment (Supplementary Figure S1A). qRT-PCR and Western blotting results showed that the mRNA and protein levels of the calcification markers Runx2, BMP2, and Osterix increased significantly in CM-stimulated VSMCs (Supplementary Figure S1B, D). Given that VSMC apoptosis via calcifying apoptotic bodies is a key driver of vascular calcification (Cui et al., 2020; Proudfoot et al., 2000), we next examined whether CM induces VSMC apoptosis. TUNEL staining results showed a significant increase in the proportion of TUNEL-positive cells after CM treatment (Figures 1D, E). The activity of caspase-3 was markedly elevated in CM-treated VSMCs (Figure 1F). These results confirmed that apoptosis is a key event in CM-treated VSMCs and directly contributes to calcification initiation.

### 3.2 Resveratrol attenuates osteogenic differentiation and calcification of rat VSMCs

**FIGURE 2:**
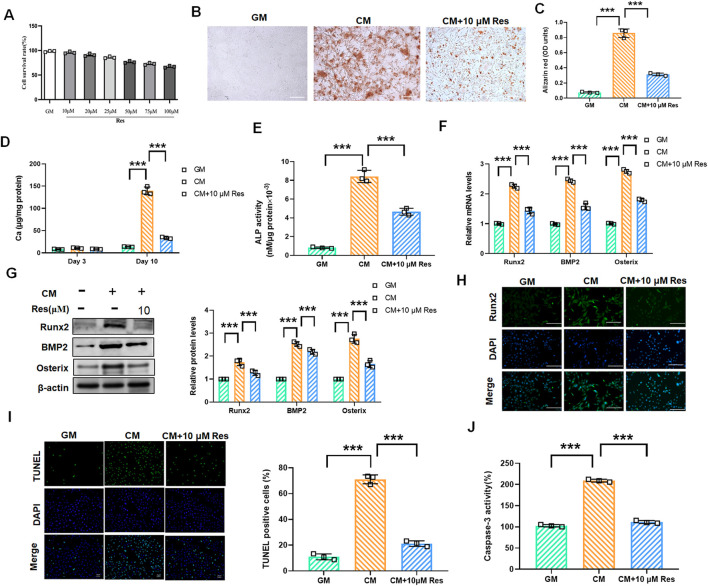
Effect of resveratrol on osteogenic differentiation and calcification in CM-treated VSMCs. VSMCs were treated with GM, CM, and/or resveratrol (10 μM), respectively. **(A)** CCK-8 assay analysis of the cell viability of VSMCs with or without resveratrol (10 μM–100 μM) treatment for 48 h. **(B,C)** Alizarin red staining and quantification of alizarin red analysis of calcium deposition in VSMCs. Scale bar = 200 μm. **(D)** Calcium content analysis of the results of calcium deposition in VSMCs. **(E)** ALP activity analysis of the results of ALP levels in VSMCs. **(F,G)** Quantification analysis of the qRT-PCR and Western blot results of Runx2, BMP2, and Osterix in VSMCs. **(H)** Representative images of immunofluorescence results of the protein levels of Runx2 in VSMCs. Scale bar = 50 μm. **(I, J)** TUNEL staining and caspase-3 activity assay analysis of cell apoptosis in VSMCs. Scale bar = 20 μm ***P < 0.001.

To assess whether Res can regulate VSMC calcification and osteogenic differentiation, we initially performed cytotoxicity assays for Res in VSMCs. After Res treatment at different concentrations, the CCK-8 assay revealed no significant alteration in VSMC viability across varying Res concentrations, indicating that Res had almost no cytotoxicity (Figure 2A). Based on these findings, we selected Res at a concentration of 10 μM for further experimental studies, which is consistent with earlier studies. Res treatment markedly reduced the deposition of calcium phosphate minerals in CM-induced VSMCs (Figures 2B, C). Calcium quantification analysis confirmed that Res treatment significantly reduced the CM-induced increase in calcium content (Figure 2D). Furthermore, we investigated the effect of Res on early osteogenic differentiation markers. The activity of ALP was significantly enhanced after CM treatment, but it decreased upon co-treatment with CM and Res (Figure 2E). Co-treatment with Res markedly suppressed the mRNA and protein levels of Runx2, BMP2, and Osterix compared to that with CM alone (Figures 2F, G). Immunofluorescence further demonstrated a significant enhancement in the fluorescence intensity of Runx2 following treatment with CM. Res effectively counteracted the above effect on Runx2 expression (Figure 2H). Importantly, Res reversed CM-induced apoptosis, as indicated by reduced TUNEL-positive cells and the decreased activity of caspase-3 (Figures 2I, J).

### 3.3 Resveratrol acts as a potential small molecule inhibitor of JNK to inhibit JNK/Bax signaling

**FIGURE 3:**
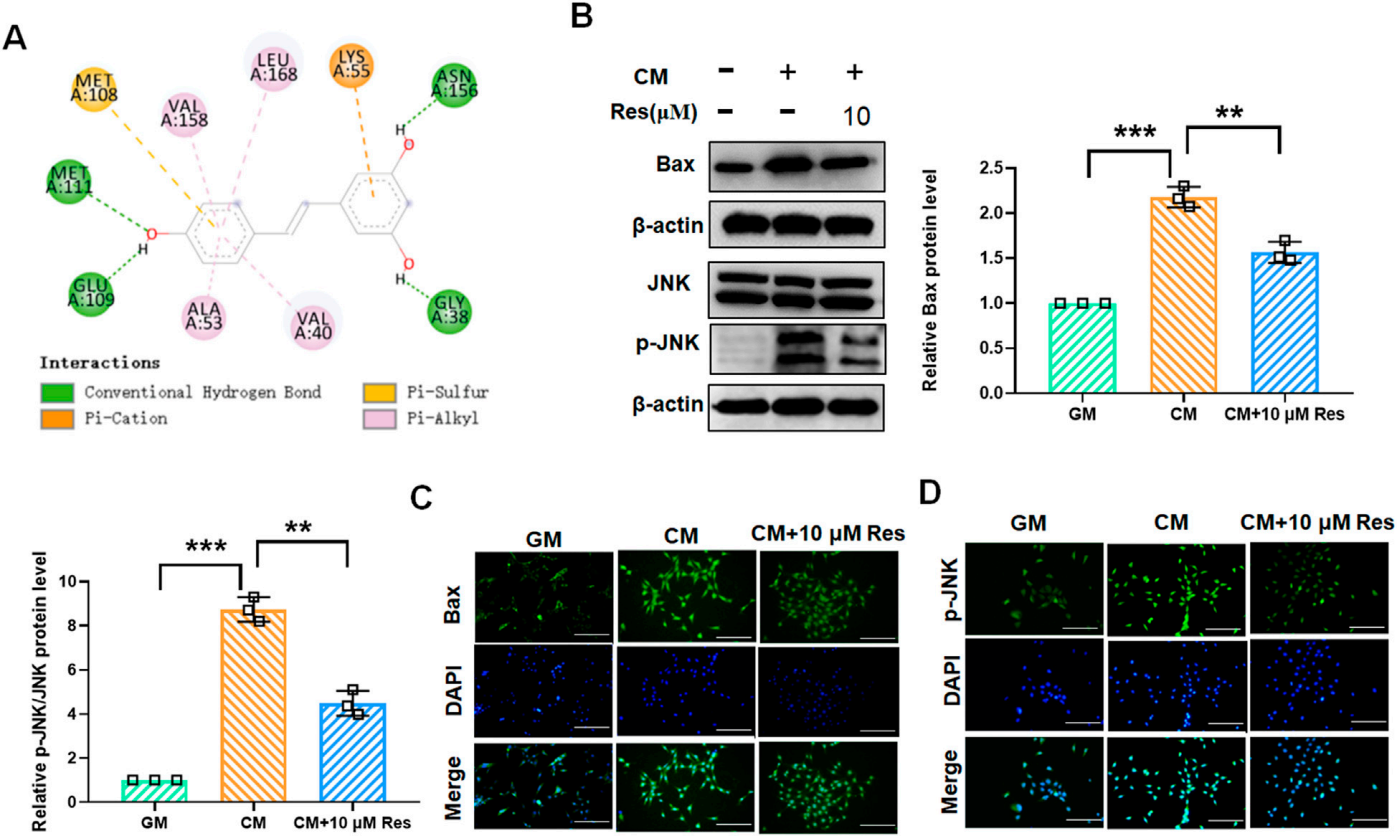
Effect of resveratrol on the JNK/Bax signaling pathway in CM-treated VSMCs. VSMCs were treated with GM, CM, and/or resveratrol (10 μM), respectively. **(A)** Docking analysis for predicting the binding mode of resveratrol to JNK. **(B)** Quantification analysis of the Western blot results of JNK, p-JNK, and Bax in VSMCs. **(C,D)** Representative images of the immunofluorescence results of the protein levels of JNK and Bax in VSMCs. Scale bar = 50 μm **P < 0.01 and ***p < 0.001.

The JNK/Bax signaling pathway is involved in the regulation of VSMC calcification and osteogenic differentiation. To explore whether Res inhibits VSMC calcification and osteogenic differentiation by regulating JNK/Bax signaling, we performed molecular docking of Res and JNK. The lowest-energy docking conformations are shown in Figure 3A, with a binding energy of −7.783 kcal/mol between Res and JNK. The docking calculations showed that Res could form four conventional hydrogen bonds with MET111, GLU109, ASN156, and GLY38 at the active center of JNK, forming a close interaction (Figure 3A). This finding indicated that Res effectively binds to and modulates JNK activity in VSMCs. Subsequently, we validated these findings using Western blot and immunofluorescence assays in CM-treated VSMCs. Western blot results confirmed that CM treatment elevated the protein levels of JNK and Bax, which was inhibited by Res co-treatment (Figure 3B). Furthermore, the results of the immunofluorescence assay demonstrated a significant enhancement in the fluorescence intensity of JNK and Bax following treatment with CM. Conversely, Res co-treatment resulted in a decrease in JNK and Bax fluorescence intensity (Figures 3C,D).

### 3.4 Inhibition of the JNK/Bax pathway attenuates osteogenic differentiation, calcification, and apoptosis of rat VSMCs

**FIGURE 4:**
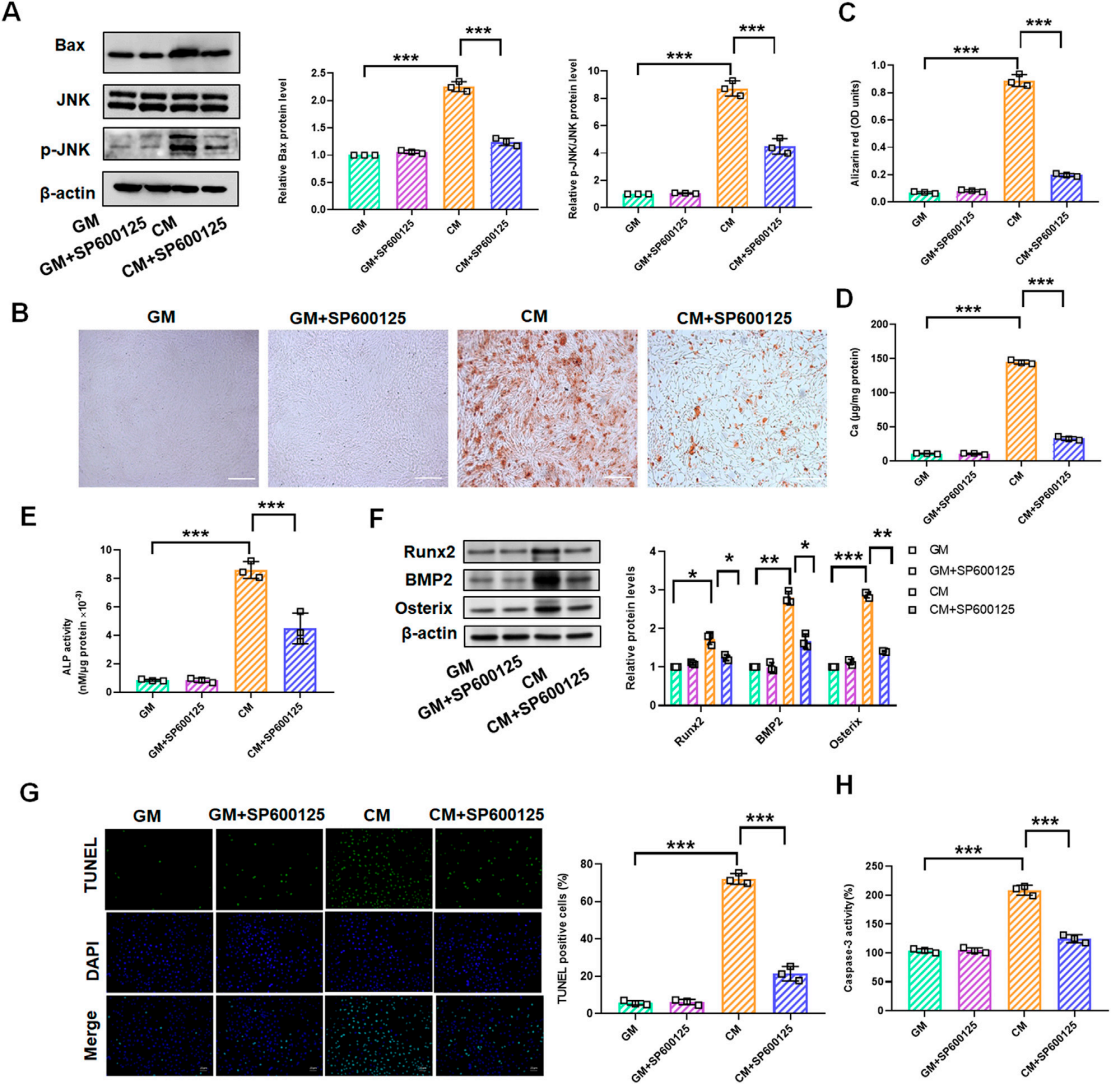
SP600125 treatment mediates the change of the JNK/Bax signaling pathways in CM-treated VSMCs. VSMCs were treated with GM and/or GM + SP600125 (10 μM) and CM and/or CM + SP600125 (10 μM), respectively. **(A)** Quantification analysis of the Western blot results of JNK, p-JNK, and Bax in VSMCs **(B,C)**. Alizarin red staining and quantification of alizarin red analysis of calcium deposition in VSMCs. Scale bar = 200 μm. **(D)** Calcium content analysis of the results of calcium deposition in VSMCs. **(E)** ALP activity analysis of the results of ALP levels in VSMCs. **(F)** Quantification analysis of the Western blot results of Runx2, BMP2, and Osterix in VSMCs. **(G, H)** TUNEL staining and caspase-3 activity analysis of cell apoptosis in VSMCs. Scale bar = 20 μm *P < 0.05, **p < 0.01, and ***p < 0.001.

To explore the role of the JNK/Bax signaling pathway in Res-mediated inhibition of vascular calcification, we treated VSMCs with CM or GM for 10 days in the presence of JNK inhibitor SP600125 (10 μM). Western blot assay revealed that CM-induced increase of JNK and Bax was markedly reduced by SP600125, and no significant difference of JNK and Bax was observed following GM and SP600125 co-treatment (Figure 4A). Alizarin red staining confirmed that SP600125 markedly prevented CM-induced calcium deposition in VSMCs (Figures 4B, C). At the same time, quantitative analysis of the cellular calcium content corroborated these findings (Figure 4D). We found that ALP activity substantially increased in the CM group but was significantly reduced following SP600125 treatment (Figure 4E). Western blotting revealed that SP600125 treatment suppressed CM-induced increases in Runx2, BMP2, and Osterix protein levels (Figure 4F). TUNEL staining and caspase-3 activity assay also demonstrated that CM-induced cell apoptosis was suppressed by SP600125 treatment (Figures 4G, H).

### 3.5 Resveratrol attenuates osteogenic differentiation, calcification, and apoptosis of rat VSMCs by the inactivation of the JNK/Bax signaling pathway

**FIGURE 5:**
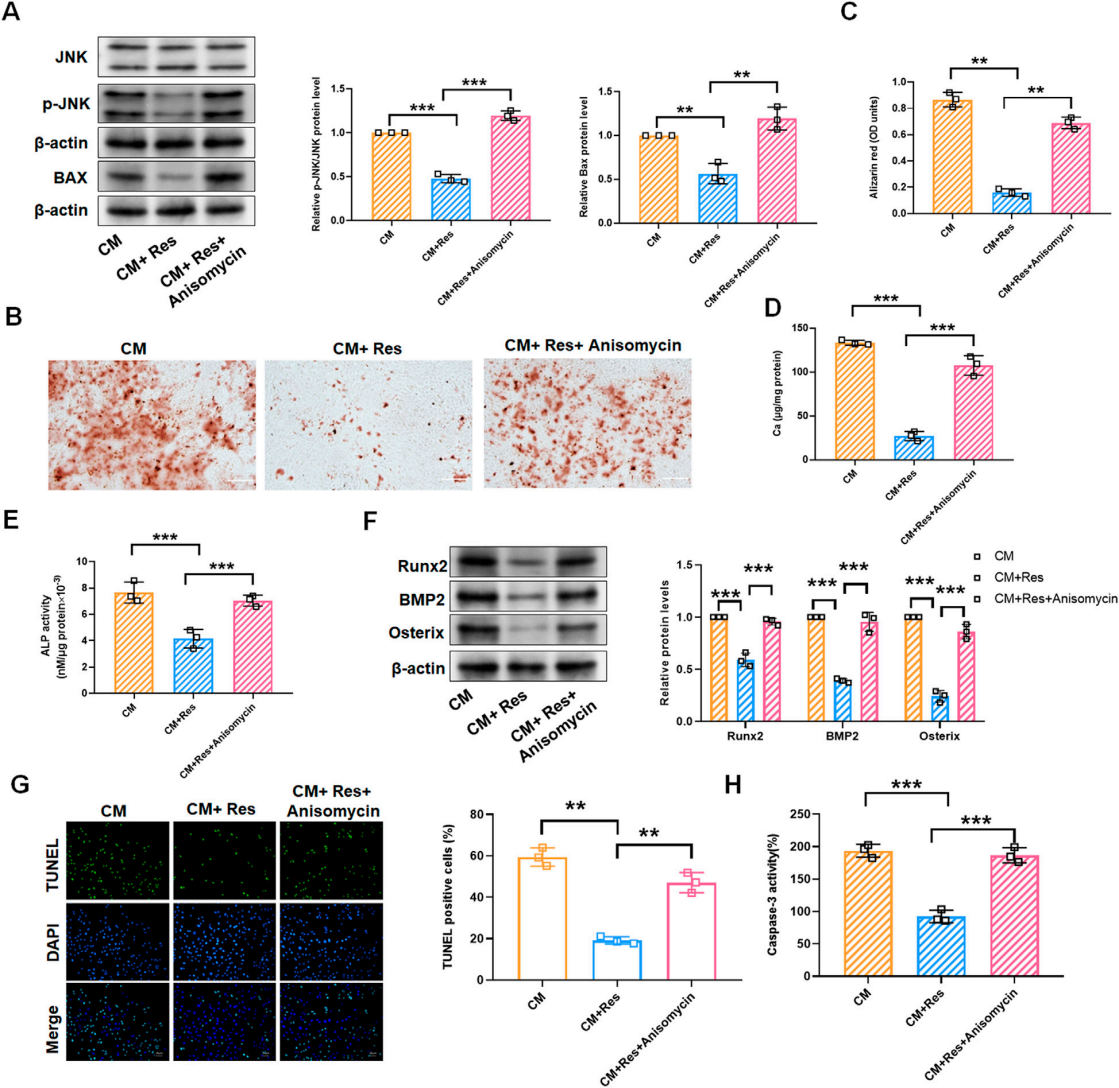
JNK/Bax pathway reverses the effect of resveratrol on CM-treated VSMC calcification and apoptosis. VSMCs were treated with CM, CM + Res (10 μM), or CM + Res + anisomycin (100 ng/mL), respectively. **(A)** Quantification analysis of the Western blot results of JNK, p-JNK, and Bax in VSMCs. **(B, C)** Alizarin red staining and quantification of alizarin red analysis of calcium deposition in VSMCs. Scale bar = 200 μm. **(D)** Calcium content analysis of the results of calcium deposition in VSMCs. **(E)** ALP activity analysis of the results of ALP levels in VSMCs. **(F)** Quantification analysis of the Western blot results of Runx2, BMP2, and Osterix in VSMCs. **(G, H)** TUNEL staining and caspase-3 activity analysis of cell apoptosis in VSMCs. Scale bar = 20 μm **P < 0.01 and ***p < 0.001.

Given the preliminary evidence of Res’s inhibitory effects on VSMC calcification and the JNK/Bax pathway, we hypothesize that Res regulates VSMC calcification by blocking the activation of the JNK/Bax pathway. As shown in Figure 5A, treatment with the JNK activator (anisomycin, 100 ng/mL) significantly reversed Res’s inhibitory effects on the expression of p-JNK and Bax proteins (Figure 5A). In CM-induced VSMCs, the downregulation of calcium deposition, calcium content, and ATP by Res was abolished upon co-treatment with anisomycin (Figures 5B–E). Similarly, the inhibitory effect of Res on the Runx2, BMP2, and Osterix levels was antagonized when Res was co-administered with anisomycin (Figure 5F). Additionally, Res treatment reduced TUNEL-positive cells and caspase-3 activity, and the combined treatment with anisomycin counteracted its effects on these parameters (Figures 5G, H).

### 3.6 Resveratrol attenuates vitamin D_3_-induced aortic calcification in mice

**FIGURE 6:**
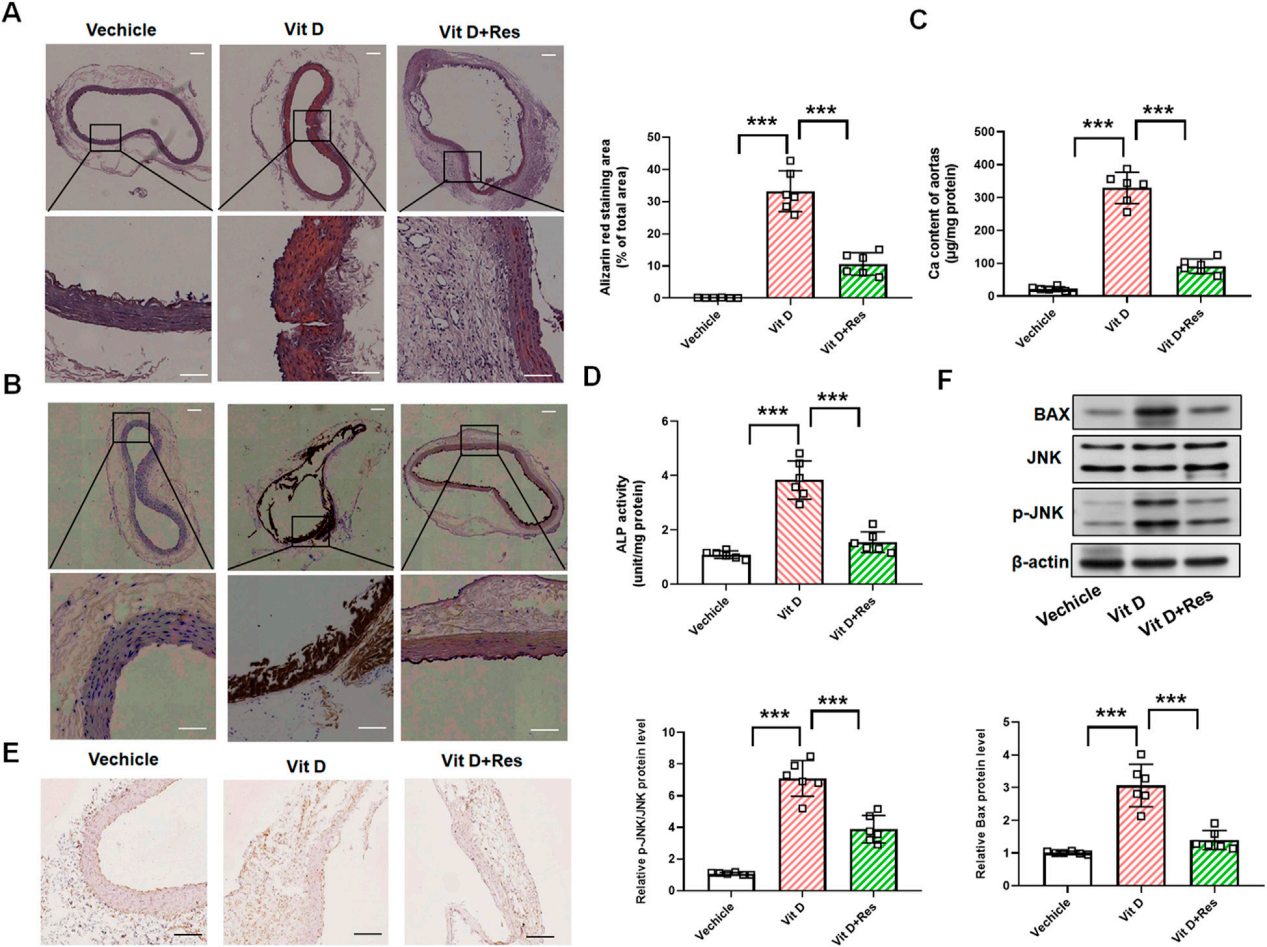
Impact of resveratrol on vascular calcification in vitamin D_3_-induced mice. **(A)** Alizarin red staining and quantification of alizarin red analysis of calcium deposition in the aortic rings. Scale bar = 100 μm; scale bar = 50 μm. (n = 6). **(B)** von Kossa staining analysis of calcium deposition in the aortic rings. Scale bar = 100 μm; scale bar = 50 μm. (n = 6). **(C)** Calcium content analysis of the results of calcium deposition in the aortas. **(D)** ALP activity analysis of the results of ALP levels in the aortic rings. (n = 6). **(E)** Representative images of immunohistochemistry results of the protein levels of Runx2 in the aortic rings. Scale bar = 100 μm. (n = 6). **(F)** Quantification analysis of the Western blot results of JNK, p-JNK, and Bax in the aortas. (n = 6). ***P < 0.001.

We further sought to determine whether Res could inhibit vascular calcification in an animal model of vascular calcification. Vascular calcification was induced in mice via subcutaneous injection of vitamin D_3_. Under the stimulation of vitamin D_3_, alizarin red staining revealed orange–red deposits, while von Kossa staining showed black–brown precipitates, indicating calcium deposition in the mouse aorta. Res intervention significantly attenuated vitamin D_3_-induced aortic calcification, as evidenced by the diminished orange–red alizarin red staining and reduced dark–brown von Kossa staining (Figures 6A, B). We lysed the mouse aortae for calcium quantification and confirmed that Res significantly inhibited the vitamin D_3_-induced increase in calcium content (Figure 6C). At the same time, we examined the activity of ALP and the expression of Runx2 as markers of osteogenic differentiation. The results revealed that Res treatment resulted in a decrease in the activity of ALP and Runx2 protein levels in mouse aorta (Figures 6D, E). Importantly, Western blot analysis demonstrated that Res treatment effectively suppressed the expressions of p-JNK and Bax in the aorta of vitamin D3-induced mice (Figure 6F).

## 4 Discussion

Vascular calcification is an active, cell-regulated process similar to bone formation, including the formation of extracellular matrix, the deposition of hydroxyphosphorus ash, and the production of osteoblast marker proteins (Shao et al., 2006). It is characterized by the transition of vascular cells, especially VSMCs, from a contractile phenotype to a synthetic phenotype. VSMCs located in the tunica media of the vascular wall induce osteogenic/chondrogenic differentiation in response to a variety of stimuli, such as calcium and phosphorus metabolism disorders, oxidative stress, DNA damage, and inflammation (Shanahan et al., 1999). In the process of transition, VSMCs secrete a variety of osteogenic differentiation marker proteins, such as Runx2, ALP, and bone morphogenetic proteins (BMPs), which promote the occurrence of vascular calcification (Li et al., 2008; Shanahan et al., 1999; Steitz et al., 2001). BMP2 can inhibit VSMC proliferation and promote VSMC apoptosis by promoting calcium and phosphorus uptake (Chen et al., 2013; Demer and Tintut, 2008). VSMCs activate vascular calcification by releasing apoptotic bodies (Liao et al., 2013; Proudfoot et al., 2000). Therefore, the phenotypic switching of VSMCs is a key mechanism to regulate vascular calcification, and effectively blocking or reversing its phenotypic switching has become the focus for the prevention and treatment of vascular calcification.

In recent years, some new drugs have been developed and used, such as vitamin D receptor agonists, calcium-sensing receptor modulators, sodium thiosulfate, and statins, which have achieved certain clinical effects in the prevention and treatment of vascular calcification, but there is still a gap with the expectations (Lunyera and Scialla, 2018; Wu et al., 2013). Res is considered a natural chemical protective agent and plays several beneficial roles in cardiovascular disease progression. Traditional animal model studies have shown that Res administration can reduce atherosclerosis and calcification in uremic mice (Tomayko et al., 2014). Related studies have shown that Res can inhibit VSMC senescence-related calcification by activating SIRT1 (Takemura et al., 2011). A cell culture study showed that Res ameliorated VSMC oxidative damage and inhibited the expression of Runx2, OPN, and HO-1; calcium deposition; and mitochondrial dysfunction (Zhang et al., 2016). In the present study, we verified the anti-osteogenic differentiation and anti-calcification effects of Res and its mechanism by establishing a mouse model of vitamin D_3_-induced aortic calcification and CM-induced VSMCs. Res treatment showed a significant reduction in calcium deposition and in ALP, Runx2, BMP2, and Osterix levels, as well as cell apoptosis in the aortic area of vitamin D_3_-induced mice and CM-induced VSMCs.

JNK is a member of the MAPK superfamily. JNK is activated by a variety of extracellular stimuli and becomes phosphorylated into the active form, which participates in VSMC apoptosis (Han et al., 2010; Zhang et al., 2014). Increasing evidence suggests that apoptosis plays an important role in the process of vascular calcification. Excessive activation of JNK can lead to apoptosis, which in turn leads to the formation of apoptotic bodies and initiates the process of vascular calcification (Liu et al., 2021; Zhou et al., 2021). In addition, several studies have revealed that JNK is an important signaling pathway regulating osteogenesis, and its activation can enhance the expression of osteogenic differentiation genes, such as Runx2 and BMP2 (Fu et al., 2019; Kim et al., 2015; Kusuyama et al., 2019; Xu et al., 2017). JNK inhibitor SP600125 can effectively reduce the apoptosis rate, ALP activity, and Runx2 and OPN expression of VSMCs (Hou et al., 2016; Liu et al., 2021; Miyazaki-Anzai et al., 2010). Here, we demonstrated that Res could activate phosphorylation of JNK and further enhance Bax expression. Sp600125 inhibited CM-induced calcium deposition, as indicated by the decreased calcium content and ALP activity; downregulated Runx2, BMP2, and Osterix expression; and reduced VSMC apoptosis. Constitutively, JNK activation removed Res’s protection against calcification and apoptosis, confirming the necessity of JNK inhibition. These data establish a causal chain from JNK inactivation to Bax suppression in Res’s mechanism of regulation of vascular calcification.

In conclusion, we demonstrated that Res could attenuate vascular calcification in VSMCs and arterial ring tissues. Mechanically, we further demonstrated that the inhibitory effect of Res on VSMC calcination was dependent on the JNK/Bax pathway to induce osteogenic differentiation and apoptosis. As a potential inhibitor of vascular calcification, this study provides new theoretical evidence for the application of Res in the early intervention and treatment of vascular calcification. Future studies employing direct functional assessments are necessary to confirm the beneficial impact of Res on the vascular physiology in the context of calcification.

## Data Availability

The original contributions presented in this study are included in the article/Supplementary Material, further inquiries can be directed to the corresponding author.
