# Supplementary figures and images for: Resveratrol ameliorates osteogenic differentiation, calcification, and apoptosis of VSMCs through regulating JNK/Bax signaling
Source: Front Pharmacol. 2025 Sep 1;16:1631039. doi: 10.3389/fphar.2025.1631039 (PMC12433885; doi:10.3389/fphar.2025.1631039)

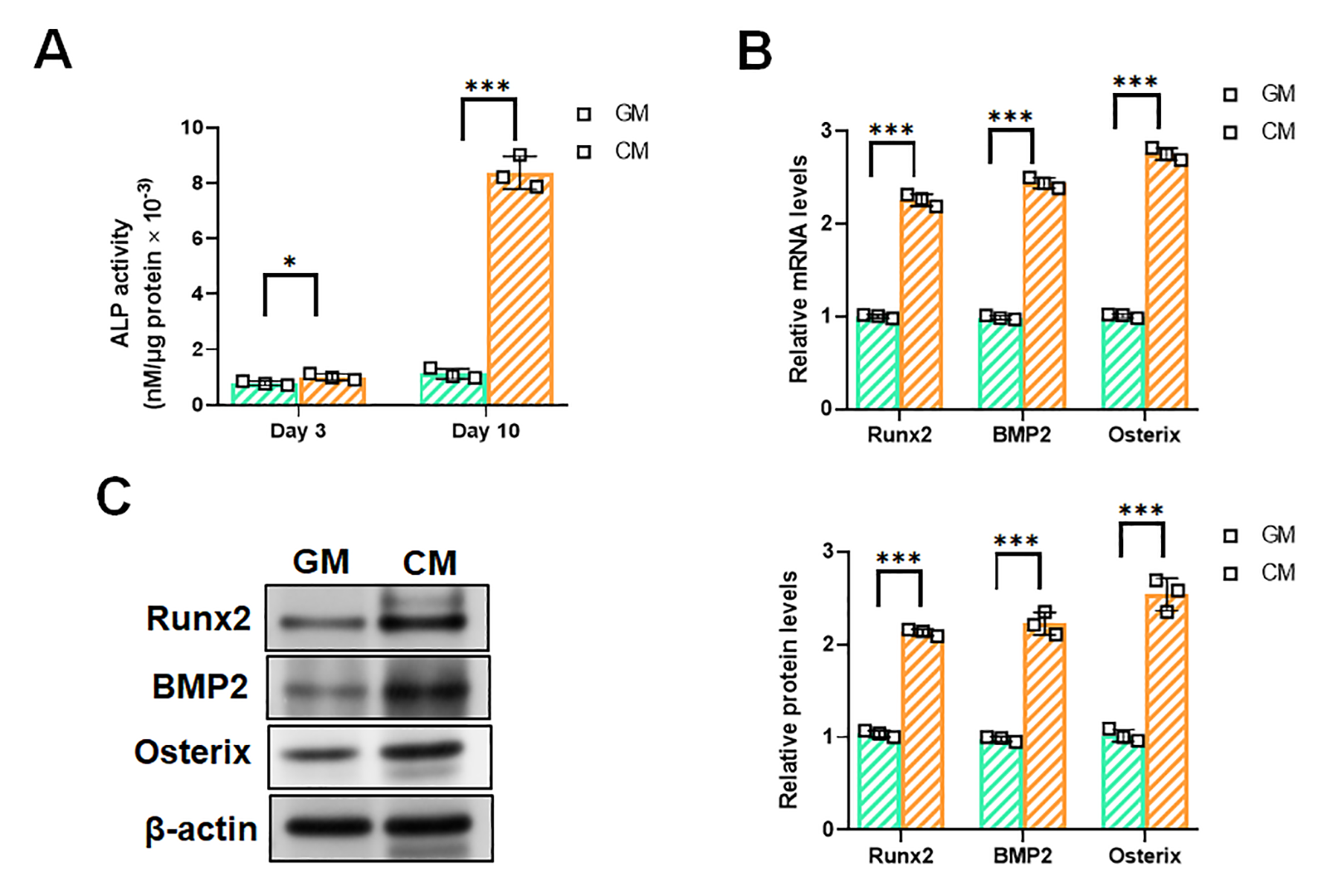

Supplement: Supplementary file 1 [file Image1.tif]
